# Thermotolerant and Thermophilic Mycobiota in Different Steps of Compost Maturation

**DOI:** 10.3390/microorganisms8060880

**Published:** 2020-06-11

**Authors:** Simone Di Piazza, Jos Houbraken, Martin Meijer, Grazia Cecchi, Bart Kraak, Ester Rosa, Mirca Zotti

**Affiliations:** 1 Laboratory of Mycology, DISTAV Department of Earth, Environmental and Life Science, University of Genoa, Corso Europa 26, 16132 Genoa, Italy; grazia.cecchi@edu.unige.it (G.C.); ester.rosa@edu.unige.it (E.R.); mirca.zotti@unige.it (M.Z.); 2 Applied and Industrial Mycology, Westerdijk Fungal Biodiversity Institute, Uppsalalaan 8, 3584 CT Utrecht, The Netherlands; j.houbraken@wi.knaw.nl (J.H.); m.meijer@wi.knaw.nl (M.M.); b.kraak@wi.knaw.nl (B.K.)

**Keywords:** food waste, harmful fungi, composting, *Scedosporium*, *Aspergillus*

## Abstract

Composting is a complex process in which various micro-organisms, mainly fungi and bacteria, are involved. The process depends on a large number of factors (biological, chemical, and physical) among which microbial populations play a fundamental role. The high temperatures that occur during the composting process indicate the presence of thermotolerant and thermophilic micro-organisms that are key for the optimization of the process. However, the same micro-organisms can be harmful (allergenic, pathogenic) for workers that handle large quantities of material in the plant, and for end users, for example, in the indoor environment (e.g., pots in houses and offices). Accurate knowledge of thermotolerant and thermophilic organisms present during the composting stages is required to find key organisms to improve the process and estimate potential health risks. The objective of the present work was to study thermotolerant and thermophilic mycobiota at different time points of compost maturation. Fungi were isolated at four temperatures (25, 37, 45, and 50 °C) from compost samples collected at five different steps during a 21-day compost-maturation period in an active composting plant in Liguria (northwestern Italy). The samples were subsequently plated on three different media. Our results showed a high presence of fungi with an order of magnitude ranging from 1 × 10^4^ to 3 × 10^5^ colony-forming units (CFU) g^−1^. The isolated strains, identified by means of specific molecular tools (ITS, beta-tubulin, calmodulin, elongation factor 1-alpha, and LSU sequencing), belonged to 45 different species. Several thermophilic species belonging to genera *Thermoascus* and *Thermomyces* were detected, which could be key during composting. Moreover, the presence of several potentially harmful fungal species, such as *Aspergillus fumigatus*, *A. terreus,* and *Scedosporium apiospermum,* were found during the whole process, including the final product. Results highlighted the importance of surveying the mycobiota involved in the composting process in order to: (i) find solutions to improve efficiency and (ii) reduce health risks.

## 1. Introduction

Composting represents one of the most efficient methods for sustainable waste management. As already highlighted by other authors, this methodology depends on many factors, the most important being the nature, composition, sizing, and quality of the substrate [1,2], environmental parameters during the process [2,3], and the microbial populations present during composting [2,4,5,6,7]. Different methodologies and technologies were applied on the basis of processed materials [1,2,6,8]. Regardless of the used technology, all composting processes reach high temperatures of about 40–55 °C degrees that are limiting and even lethal for many organisms. The high temperatures of the composting process create strong selection on the biological components: on the one hand, it eliminates many mesophilic organisms that are threatening for plants, but selects for extremophilic and extremotolerant organisms particularly adapted to high temperatures. These thermophilic and thermotolerant micro-organisms are fundamental in biochemical processes since several species have key roles for their effectiveness in lignin degradation and thermoresistant- or thermostable-enzyme production. However, potentially dangerous propagules of the same organisms can be inhaled, becoming harmful for workers who handle daily large quantities of compost, and for end users that live or work, especially in indoor environments, near potentially contaminated sources (e.g., potted plants). Therefore, knowledge of the microbiological components of compost is essential to optimize the process and to make a product of excellent quality, and free of hazards for workers, consumers, and the environment.

In the last few decades, some authors studied the microbial components of compost, mainly focusing on the bacterial components [7,9,10,11], and other studies listed potentially dangerous species for humans [12,13]. Thanks to these studies, commercial products with special bacterial starter cultures, endomycorrhizal fungi, and chemical activators (in particular nitrogen) were developed, and are useful both on the domestic and industrial scale, resulting in the improvement of the composting process and lowering production costs.

With the advent of new high-throughput sequencing technologies, nonculture-based approaches were applied to characterize microbial communities, giving a wider picture on them than classical culture-based approaches [7,9,12,13,14,15]. These methods provided excellent indications of the community, but did not allow the isolation of organisms involved in the process. For these reasons, a culture-dependent method is still essential to isolate vital strains present in the matrix. In fact, isolation of the organisms into an axenic culture allows: (i) to identify organisms, safely discriminating potentially dangerous from harmless species; (ii) verification of the effective viability of strains present in the investigated matrix; (iii) isolation and preservation of living organisms; and (iv) study and exploitation of the organisms’ biochemical characteristics.

In the last decade, studies investigated the fungal communities in compost, confirming that fungi are a significant part of the microbial community, in particular during the maturation phase [1,8,14,16,17,18,19,20,21,22,23]. Moreover, some of these studies detected fungal species that are useful during stages of maturation [19,21,24] and/or potentially harmful [12,17,20,25]. For these reasons, it is fundamental to conduct studies on fungal communities involved in the composting processes in order to select both useful populations to improve the process and to investigate the presence of potentially harmful species.

This work contributes to the knowledge of thermotolerant and thermophilic fungal communities in compost. We characterized the fungal community of compost samples taken during the ripening of the product at five different stages in a composting plant in northwestern Italy. After isolation of the strains in pure culture, they were identified at the species level using the latest taxonomic insights.

## 2. Materials and Methods 

### 2.1. Composting Procedure and Sampling

Samples were collected from a compost-production plant in Liguria (Cairo Montenotte, northwestern Italy). The plant produces biogas through anaerobic digestion in a batch system from the organic fraction of urban solid waste (OFMSW) collected from a municipality in the area. After the anaerobic-digestion phase, a new mixture is made composed of 1:1 (*w:w*) by the anaerobic digestate (which has a pH ranging between 6.5 and 7.5, and a temperature of 55 °C), and green and brown waste from municipal green planting and pruning. This new mixture is composted in chambers measuring 4 m × 8 m × 2 m. Ripening time is, on average, 21 days after which the product is ready to leave the plant.

As part of this study, the pile was sampled at five different times during the ripening process to analyze the mycological components present and their evolution over time. More precisely, the biodigestate (sample TQ) was analyzed, and samples were collected after 1, 7, 14, and 21 days of maturation (samples 1D, 7D, 14D, 21D). Given the size of the chambers, each sample consisted of 10 randomly taken composite samples of the biopile and biodigestate at various stages of maturation. 

### 2.2. Fungal Isolation

Fungi were isolated by a modified plate dilution method [26]. Each solid sample was initially diluted 1/10 in sterile water, and the suspension was shaken at room temperature for 1 min. Later, the suspension was further diluted 1/100 and 1/1000. The three dilutions were plated out on three different media (9 cm ⌀ Petri dishes) in triplicate: malt-extract agar supplemented with penicillin and streptomycin (MEA/PS), oatmeal agar with penicillin and streptomycin (OA/PS), and dichloran 18% glycerol agar (DG18). The plates were incubated at four temperatures (25, 37, 45, and 50 °C) and checked daily between 3 and 15 days. Colonies were counted, and these data were used to determine the concentration of fungi (number of colony-forming units per gram, CFU/g) in the compost. For longer-term studies, the isolates were stored at −20 °C in the Laboratory of Mycology DISTAV University of Genoa (ColD) collection, and in the working collection of the Department of Applied and Industrial Mycology housed at the Westerdijk Fungal Biodiversity Institute, Utrecht, the Netherlands (DTO culture collection).

### 2.3. Molecular Identification

A preliminary sorting of the isolated strains was carried out on the basis of phenotypic characters. DNA barcoding of the isolated strains was carried out by amplification of the internal-transcribed-spacer (ITS) region using primer pair V9G and LS266 [27]. Later, in order to obtain more precise identification, other fragments were amplified on the basis of the (preliminary) identification result based on ITS sequencing (beta-tubulin for *Penicillium*, calmodulin for *Aspergillus*, elongation factor 1-alpha, and LSU for others) according to Samson et al. [27]. In short, genomic DNA was extracted from 3-day old cultures grown on MEA, using the Qiagen DNeasy Ultraclean™ Microbial DNA Isolation Kit (Qiagen, Hilden, Germany) according to manufacturer instructions. The PCR reaction contained 12 μL mix (8.23 μL MillQ water, 0.38 μL 50 mM MgCl_2_, 1.25 μL NH 10× PCR buffer taq, 0.98 μL 1 mM dNTPs, 0.63 μL DMSO, 0.25 μL 10 µM of each primer, 0.05 BioTaq 5 U/µL) and 1 μL of DNA template. The PCR program was: 5 min 95 °C, 30–35 × (35 s 95 °C, 50 s 55 °C, 2 min 72 °C), 5 min 72 °C, 10 °C for ∞. Amplicons were bidirectionally sequenced, assembled and compared using BLAST (Basic Local Alignment Search Tool) against sequences present in the sequence database of the National Center of Biotechnology Information (NCBI, http://blast.ncbi.nlm.nih.gov/Blast.cgi) (GenBank) and sequences present in the internal database of the Westerdijk Institute.

### 2.4. Data Analysis

Fungal-biodiversity level (H’) was calculated for each sample with Shannon’s biodiversity index [28,29,30]. Related evenness (E) was expressed as the ratio between H’ and the maximal potential biodiversity of each sample (HMAX). HMAX was expressed as the logarithm base 2 of the total number of observed species. The Jaccard similarity coefficient [31], calculated as the ratio of intersection and union of each pair of samples, was studied to evaluate the affinity between the isolated strains at the time of sampling. All indices and graphs were made in a Microsoft Excel spreadsheet.

## 3. Results

The heat map in Table 1 represents the amount of CFUs observed during the monitoring period at different incubation temperatures. The order of magnitude observed of CFUs per gram of compost rose from 1 × 10^4^ CFU g^−1^ to 3 × 10^5^ CFU g^−1^ during a maturation period of 21 days.

The isolated strains belonged to 25 genera and were identified as 45 different species. The obtained sequences were deposited in GenBank with the following accession numbers: MT316336-MT616380, MT312848-MT312857, MT420412-MT420424, and MT433447-MT433470. The heat map in Table 2 reports the taxonomic assignment of the isolated strains and their abundance (calculated as CFU per gram of compost) observed in each site at each time, and their related GenBank accession numbers. The heat map gives an overview of the occurrence of different strains during the maturation period, highlighting the presence of some species in several steps of maturation. The most frequently occurring fungal species (isolated in all five samples) were *Scedosporium apiospermum, Thermomyces dupontii* (=*Talaromyces thermophilus*) and *Thermomyces lanuginosus*. Strains of *Aspergillus chevalieri, A. terreus,* and *Talaromyces trachyspermus* were isolated in 4 of the 5 samples. They were followed by six species that were isolated in three of the five samples: *Aspergillus fumigatus, Geotrichum* sp., *Malbranchea cinnamomea*, *Rasamsonia emersonii* (=*Talaromyces emersonii*), *Scedosporium aurantiacum,* and *Scopulariopsis brevicaulis.*

**Figure 1:**
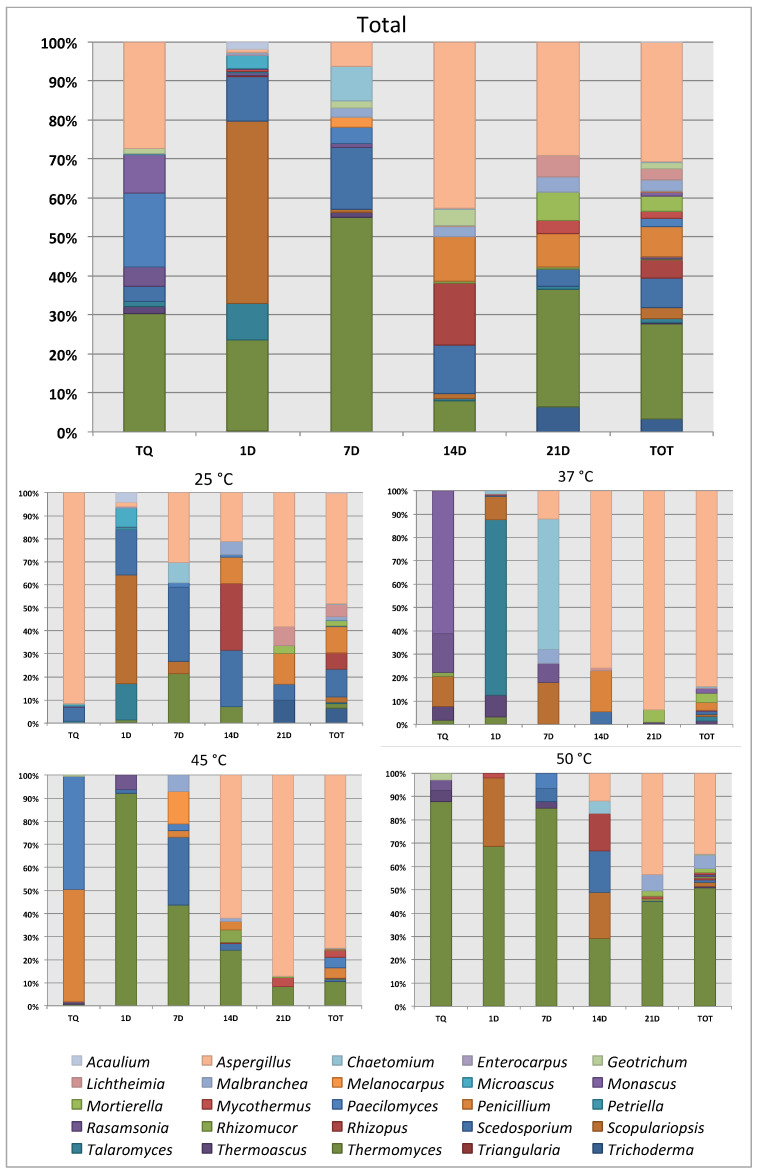
Percentage distribution by genera of filamentous fungi isolated during maturation period: total and detail for each temperature of incubation.

Figure 1 reports the total generic distribution and the related distribution at each temperature, expressed as observed percentage in the samples during the maturation period. The cumulative Shannon’s index of all samples, as shown in Table 3, ranged from 2.60 to 3.44, and evenness increased from 0.67 to 0.84 during the maturation process, highlighting equality in the level of distribution of individuals among the various species present in the mature samples. Results about the assessment of biodiversity at the different temperatures are also reported in Table 3.

Table 4 reports the results for Jaccard specific similarity between the analyzed samples. During the maturation period, the Jaccard value was higher between adjacent samples while samples distant over time were more different.

## 4. Discussion

Composting is a complex process based on the biodegradation of organic matter in which fungi play a key role during the ripening phase. Our data, collected in an active plant, confirmed the presence of thermotolerant and thermophilic fungi. During the maturation period, the number of CFUs increased, biodiversity rose, while mycobiota evolved from unequal distribution among species at the beginning of maturation to fairer distribution between species at maturity. Despite this evolution, we confirmed that the mycobiota were rather stable from a qualitative point of view. However, several potentially pathogenic species emerged in all phases of the cycle, and, for this reason, the periodic monitoring of the concentrations of certain species during the production cycle is necessary. For example, in our case, the opportunistic pathogenic fungal species *Aspergillus fumigatus*, *A. terreus,* and *Scedosporium* spp (in particular *S. apiospermum*) were found in high concentrations.

Analyzing our data, we observed an increase in the number of CFUs during the maturation period up to 10 times in the final product compared to the digestate and the mixed product sampled after one day of maturation (see Table 1). The high temperatures at the beginning of the process probably temporarily inhibited germination that restarted once the pile reached a temperature of around 45 °C.

With regard to the specific composition of the sampled populations, about 25% of the isolated strains were shared in at least 3 of the 5 samples, thus present in at least 60% of the process (Table 2). The Jaccard index shown in Table 3 indicates a similarity between samples ranging from 0.16 to 0.46. The Jaccard index reported in Table 4 shows that the similarity between successive samples increased over time, and the specific composition of the sample became more stable. Furthermore, Figure 1 shows that, in the first four samples (TQ, 1D, 7D, 14D), there was a strong predominance of genera *Thermomyces*, *Scedosporium,* and *Aspergillus,* while the mature sample (21D) was clearly more homogeneous. This was further confirmed by the diversity indices shown in Table 2. In fact, the biodiversity index rose during maturation from 2.60 to 3.44. More interesting is the trend of evenness that rose steadily to reach a value of 0.84 in the mature sample. This confirmed that, with the progress of the maturation process, distribution in the species within the community became increasingly homogeneous and balanced.

Results concerning the isolated fungi are consistent with previously published data on compost samples [17,18,19,21]. The main differences could be traced back to changed taxonomic classifications. For example, key species *Thermomyces dupontii* was, until 2014, known under its old name, *Talaromyces thermophilus* [32]. Other important compost-associated species that underwent name changes are *Rasamsonia emersonii* and *Mycothermus thermophilus*, previously known as *Talaromyces emersonii* and *Scytalidium thermophilum*, respectively. The isolates were primarily identified using ITS sequencing. However, this locus lacks resolution at the species level for some genera detected in the compost, such as *Aspergillus*, *Penicillium*, *Scedosporium*, *Scopulariopsis,* and *Talaromyces*. In order to obtain reliable species identifications, additional genes (so-called secondary barcodes) were generated to confirm the presence of key species in the composting process. Because information is often linked to a species, correct identification is important from a biotechnological and medical point of view. Accurately identified isolates in this study could have added value for metagenome studies where only partial ITS sequences are often used.

We isolated several thermophilic fungi capable of producing enzymes such as amylase, xylanase, phytase, and chitinase, among which were *Thermomyces dupontii* (=*Talaromyces thermophilus*), *Thermomyces lanuginosus*, and *Thermoascus aurantiacus* that degrade the woody components in the compost. However, opportunistic pathogenic species were also found in the samples. The most recurrent genus was *Scedosporium*. In particular, *Scedosporium apiospermum* was found in all samples, including the mature compost. Five other species, *S. aurantiacum*, *S. brevicaulis*, *S. dehoogii, S. minutisporum,* and *S. prolificans,* were found in decreasing numbers. Moreover, a strong presence of *Aspergillus fumigatus* was detected in the mature samples. The high temperatures, therefore, shaped the mycobiota during compost maturation. Only some specialized species can survive and proliferate in these particularly hostile conditions. Our results agreed with those of López González et al. [19]. In fact, we found some predominant species throughout the process (resident mycobiota) in contrast to others occasionally found (transient mycobiota).

## 5. Conclusions

This is one of few studies that analyzed mycobiota during compost-maturation phases, and is currently the only one in which species were accurately identified using ITS sequencing supplemented with secondary barcodes. Our results confirmed high biodiversity of the fungal component that tends to stabilize during the compost-maturation process. Results showed a high presence of thermophilic and thermotolerant species that, if properly managed, could improve the composting process. However, a significant presence of species potentially harmful to the health of workers and end users should also be noted.

## Figures and Tables

**Table 1:** Heat map reporting of colony-forming units (CFU) g^−1^ in each site for each temperature.

		Samples				
		TQ	1	7	14	21
Incubation Temperature	25 °C					
	37 °C					
	45 °C					
	50 °C					

Scale bar ×10^4^ CFU g^−1^
**1**





**30**

**Table 2:** Heat map showing isolated species and their abundance (calculated as CFU g^−1^ of compost), at each observed time. Their related GenBank accession numbers are reported; - means that the secondary barcode is not available.

	Samples					GenBank Accession Numbers	
Species	TQ	1D	7D	14D	21D	ITS Barcode	Secondary Barcode
*Acaulium acremonium*						MT316336	MT312849, MT433447
*Aspergillus chevalieri*						MT316337	MT420413
*Aspergillus fumigatus*						MT316338	MT420414
*Aspergillus nidulans*						MT316339	MT420415
*Aspergillus niger*						MT316340	MT420412
*Aspergillus pseudoglaucus*						MT316341	MT420416
*Aspergillus quadrilineatus*						MT316342	MT420417
*Aspergillus terreus*						MT316343	MT420418
*Aspergillus sp.*						MT316344	MT420419
*Chaetomium thermophilum*						MT316345	MT433448
*Chaetomium uniapiculatum*						MT316346	MT433449
*Enterocarpus grenotii*						MT316347	MT312850
*Geotrichum* sp.						MT316348	MT312851
*Lichtheimia corymbifera*						MT316349	MT312852
*Malbranchea cinnamomea*						MT316350	MT312853
*Melanocarpus albomyces*						MT316351	MT433450
*Microascus paisii*						MT316352	MT433451
*Microascus restrictus*						MT316353	MT433452
*Monascus ruber*						MT316354	MT433453
*Mortierella wolfii*						MT316355	MT312854
*Mycothermus thermophilus*						MT316356	MT433454
*Paecilomyces variotii*						MT316357	MT433455, MT312848
*Penicillium crustosum*						MT316358	MT433456
*Penicillium* sp. 1						MT316359	-
*Penicillium* sp. 2						MT316360	-
*Petriella* sp.						MT316361	MT433457
*Rasamsonia emersonii*						MT316362	MT433458
*Rasamsonia* sp.						MT316363	MT433459
*Rhizomucor miehei*						MT316364	MT312855
*Rhizopus microsporus*						MT316365	MT312856
*Rhizopus oryzae*						MT316366	MT312857
*Scedosporium apiospermum*						MT316367	MT433460
*Scedosporium aurantiacum*						MT316368	MT433461
*Scedosporium dehoogii*						MT316369	MT433462
*Scedosporium minutisporum*						MT316370	MT433463
*Scedosporium prolificans*						MT316371	MT433464
*Scopulariopsis brevicaulis*						MT316372	MT433465, MT420423
*Talaromyces trachyspermus*						MT316373	MT433466
*Talaromyces tratensis*						MT316374	MT420420
*Talaromyces wortmannii*						MT316375	MT420421
*Thermoascus aurantiacus*						MT316376	MT433467, MT420422
*Thermomyces dupontii*						MT316377	MT433468
*Thermomyces lanuginosus*						MT316378	MT433469
*Triangularia* sp.						MT316379	MT433470
*Trichoderma longibrachiatum*						MT316380	MT420424

Scale bar ×10^4^ CFU g^−1^
**0**










**10**

**Table 3:** Shannon’s biodiversity index (H’) and evenness (E) of analyzed samples detailed at different temperatures, and cumulative samples (TOT).

		TQ	1D	7D	14D	21D
**25 °C**	**H’**	0.69	2.56	2.83	2.91	2.70
	**E**	0.22	0.67	0.89	0.84	0.90
**37 °C**	**H’**	1.69	1.41	2.21	1.96	2.14
	**E**	0.65	0.44	0.74	0.70	0.76
**45 °C**	**H’**	0.33	1.15	2.12	1.61	1.96
	**E**	0.14	0.58	0.82	0.57	0.70
**50 °C**	**H’**	0.72	1.45	1.12	2.82	1.77
	**E**	0.36	0.72	0.48	0.94	0.53
**TOT**	**H’**	2.60	2.67	2.65	3.39	3.44
	**E**	0.67	0.62	0.65	0.77	0.84

**Table 4:** Jaccard’s similarity index.

	TQ	1D	7D	14D	21D
**TQ**	1	0.25	0.45	0.24	0.19
**1D**		1	0.28	0.24	0.16
**7D**			1	0.36	0.21
**14D**				1	0.46
**21D**					1
